# Arbuscular Mycorrhizal Fungi and Exogenous Calcium Synergistically Alleviate Arsenic Stress in Cotton Seedlings

**DOI:** 10.3390/jof12050335

**Published:** 2026-05-04

**Authors:** Qiaoming Zhang, Wenjing Yang, Caiyun Zhang, Lirong Ren, Na Bai, Lin Zhang, Chen He, Minggui Gong

**Affiliations:** 1 College of Horticulture and Plant Protection, Henan University of Science and Technology, Luoyang 471023, China; zhangqm1013@163.com (Q.Z.); ywenjing0517@126.com (W.Y.); zhagln20@126.com (L.Z.); chenhehaust@126.com (C.H.); 2 College of Food and Bioengineering, Henan University of Science and Technology, Luoyang 471023, China; 18326212657@163.com (C.Z.); 15937715759@163.com (L.R.); zyg220422@163.com (N.B.)

**Keywords:** cotton, arsenic stress, arbuscular mycorrhizal fungi, exogenous calcium, ionic homeostasis

## Abstract

Cotton (*Gossypium hirsutum* L.) is a promising candidate for an Arsenic (As)-tolerant plant due to its low As accumulation in fibers. The individual arbuscular mycorrhizal fungi (AMF) inoculation or exogenous calcium (Ca^2+^) application is known to enhance heavy metal tolerance in higher plants; however, their synergistic mechanisms in alleviating As stress in cotton remain poorly understood. A three-factor pot experiment was conducted, including two levels of AMF (*Funneliformis mosseae* C.Walker & A.Schüßler) inoculation (non-inoculated/inoculated), As stress (0/100 mgAs^5+^·kg^−1^soil), and exogenous Ca^2+^ (CaCl_2_) application (0/20 mmol·L^−1^ CaCl_2_). AMF inoculation and Ca^2+^ application were investigated for their effects on cotton growth, root morphology, photosynthetic characteristics, osmotic regulators, antioxidant enzyme activities, and ion homeostasis under As stress. Results showed As stress significantly disrupted cotton growth (decreased plant height, shoot and root dry weight) and root morphology (reduced total root length, root area, and root fork number), photosynthetic capacity (reduced *Pn*, *Ci*, *Fv/Fm*, and *ΦPSII*), osmotic adjustment (decreased proline, soluble sugar and protein), antioxidant defense (inhibited SOD, POD, CAT activities), and K^+^/Ca^2+^ homeostasis (reduced concentration of K^+^ and Ca^2+^, and K^+^/Ca^2+^ ratio). Both AMF inoculation and Ca^2+^ application independently alleviated these adverse effects of As stress. At the same time, AMF symbiosis combined with exogenous Ca^2+^ was better than AMF inoculation or Ca^2+^ application alone in optimizing root architecture, improving stomatal function and photosynthetic efficiency, enhancing osmotic regulator accumulation and antioxidant enzyme activities, and restoring ion balance under As stress. Three-way ANOVA confirmed significant As×AMF×Ca^2+^ interactions on key parameters such as *Pn* and *ΦPSII*. In summary, both AMF inoculation and Ca^2+^ application synergistically enhanced cotton As tolerance through regulating growth, root morphology, photosynthetic characteristics, osmotic regulators, antioxidant enzyme activities, and ion homeostasis, demonstrating its potential for sustainable cotton cultivation in As-contaminated soils.

## 1. Introduction

Arsenic (As) is a ubiquitous and highly toxic heavy metal that poses a severe threat to agricultural ecosystems and global food security [1,2]. It is released into the environment via a variety of natural processes, such as rock weathering, volcanic emissions, and discharge from hot springs [3]. As accumulation in soil ecosystems has become a widespread environmental problem, caused by various anthropogenic activities such as As ore mining, metal smelting, fossil fuel combustion, and the application of As-containing agrochemicals, herbicides, wood preservatives, and phosphate fertilizers [4]. Inorganic arsenic is classified as a Group I carcinogen, owing to its bioaccumulation in the food chain and elevated concentrations in drinking water. Chronic As poisoning has become an increasingly severe problem in Asia, South America, and other regions. Inorganic As(III) and As(V) in soil and water are easily absorbed by plants, due to stronger adhesion and mobility [2]. Its accumulation in plants disrupts tissues’ key physiological, biochemical and growth processes (such as nutrient uptake, photosynthesis, and redox homeostasis), causing growth inhibition, yield reduction, senescence and even death, which ultimately triggers vegetation degradation in As-contaminated regions [5]. To cope with arsenic stress, numerous plant species have evolved various adaptive strategies to improve their As tolerance, which lays a foundation for the safe utilization of As-contaminated land and the promotion of sustainable agricultural development.

Cotton (*Gossypium hirsutum* L.), a globally important cash crop widely cultivated in agroecosystems with diverse climates, is highly susceptible to heavy metal stress. It has emerged as a promising candidate for the As-tolerant plant in As-contaminated soils, primarily due to its low As accumulation in fibers, large biomass production, strong stress resistance, and high economic value [6,7]. However, excessive As accumulation in cotton tissues still poses a serious threat to growth and physiological metabolic processes [8]. High concentrations of cotton not only inhibit plant growth and photosynthetic characteristics, but also induce ionic imbalance, osmotic stress, and oxidative damage [8,9]. There is an urgent need to develop sustainable strategies to enhance cotton’s tolerance to arsenic stress. In recent decades, the use of beneficial soil microorganisms and mineral nutrients has been recognized as an environmentally friendly and sustainable approach to alleviate heavy metal toxicity in plants [10].

Arbuscular mycorrhizal fungi (AMF) are ubiquitous symbiotic soil microbes that form mutualistic associations with more than 80% of terrestrial plants, and this symbiosis is mainly achieved by colonizing root cortical tissues to produce arbuscules, hyphae and vesicles [11]. Serving as a critical “bridge” between host plants and rhizosphere soil, AMF acquires mineral nutrients (including phosphorus, nitrogen, and various micronutrients) via arbuscule and extraradical hyphae, subsequently transferring these to the host plant, while receiving 5–15% of the host plant’s photosynthetic carbohydrates and lipids from the host plant [12,13]. AMF hyphae sequester heavy metals in fungal structures to reduce their translocation to shoots [10]. AMF also enhances antioxidant enzyme activities and total phenolic content, thereby alleviating oxidative damage and ultimately improving photosynthetic capacity and plant growth under heavy metal stress conditions [4]. Previous studies have demonstrated that AMF symbiosis enhances plant resistance to As stress. *Rhizophagus intraradices* alleviates As toxicity in *Robinia pseudoacacia* by promoting plant growth, optimizing root morphology, regulating phytohormones, and increasing soil glomalin concentration [4]. Furthermore, *R. intraradices* enhances As tolerance in *Sophora davidii* by improving growth, promoting gas exchange, regulating reactive oxygen species levels and antioxidant enzyme activities, while reducing As accumulation in *S. davidii* tissues, while reducing As accumulation in *S. davidii* tissues [14]. Calcium-dependent protein kinases (CDPKs function as “sensor responders”, directly decoding Ca^2+^ signatures into phosphorylation-mediated signaling cascades [15]. AMF inoculation upregulates 11 calcium-dependent protein kinase (CDPK) genes, and downregulates 8 *PtCDPKs* in *Populus tomentosa* under As stress, indicating a regulatory role of AMF in *PtCDPK*-mediated stress responses [13].

Calcium (Ca) is recognized not only as an essential macronutrient for plant growth and development, but also as a highly conserved secondary messenger involved in sensing, transducing, and responding to external environmental signals [16]. It serves as a key regulatory factor governing plant cell metabolism, signal transduction, photomorphogenic development, and abiotic/biotic stress responses [17]. Exogenous Ca^2+^ application has been proven to alleviate heavy metal toxicity through re-establishing cellular ionic, osmotic, and reactive oxygen species (ROS) homeostasis [16,18]. Ca^2+^ maintains cell membrane stability by reducing lipid peroxidation [9]. It regulates ion homeostasis by competing with heavy metals for binding sites on the plasma membrane [19], and modulates the activity of antioxidant enzymes and osmotic regulators [20]. Previous studies have demonstrated that Ca^2+^ application effectively mitigates Cd stress in sesame [21] and chickpea [9], and salt stress in peanut [22] by enhancing photosynthetic capacity, elevating the antioxidant enzymes’ activities, and restoring ionic balance. Additionally, Ca^2+^ has been shown to promote AMF colonization and symbiosis development, suggesting potential synergistic interactions between AMF inoculation and Ca^2+^ application in stress tolerance [16,22]. AMF symbiosis increases Ca^2+^ content in peanut seedlings, while exogenous Ca^2+^ application in turn promotes AMF colonization in peanut roots. Both AMF inoculation and Ca^2+^ application effectively alleviate salt stress in peanut [16,22]. Although extensive studies have investigated the individual effects of AMF inoculation and Ca^2+^ application on alleviating environmental stress, their synergistic mechanisms underlying arsenic stress tolerance in plants remain largely unclear. The interactive effects of AMF inoculation and Ca^2+^ application on As-stressed cotton—including their impacts on root architecture, photosynthetic efficiency, antioxidant defense, and ion homeostasis—have not been systematically investigated.

The present study is designed to investigate the synergistic effects of AMF and exogenous CaCl_2_ on cotton tolerance to As stress. A pot experiment with a three-factor randomized complete block design is conducted on cotton seedlings. The treatments included AMF inoculation or non-inoculation, Ca^2+^ application or no Ca^2+^, and exposure to non-As or As stress conditions. Plant growth, root morphology, photosynthetic characteristics, osmotic regulators, antioxidant enzyme activities, and ion homeostasis are systematically measured. This study aims to (1) evaluate the individual and combined effects of AMF inoculation and exogenous Ca^2+^ application on alleviating As-induced damage in cotton seedlings; (2) elucidate the underlying synergistic mechanisms involved in growth promotion, photosynthetic optimization, stress defense, and ion homeostasis; (3) provide a theoretical basis and practical, environmentally friendly strategy for improving cotton productivity in As-contaminated soils. The present study will advance our understanding of the synergistic effects of AMF and exogenous CaCl_2_ on alleviating As stress in cotton seedlings, and will elucidate the physiological regulatory mechanisms by which their combination enhances As tolerance in cotton seedlings.

## 2. Materials and Methods

### 2.1. Experimental Design, Biological Material, and Growth Conditions

The experiment consisted of a randomized complete block design with three factors. The first factor was AMF inoculation treatment, which comprised two levels of AMF inoculation: Non-AMF (NM) and AMF-inoculation (AM), the second factor was As stress treatment, including two levels of As stress condition: non-As (As_0_) and As (As_100_) stress, and the third factor was exogenous Ca^2+^ application treatment, comprised two levels of exogenous Ca^2+^ application: (Non-Ca^2+^ (Ca^2+^_0_) and Ca^2+^ (Ca^2+^_20_) application) were applied in this experiment. The experiment consistedof the following treatments: (1) NM + As_0_ + Ca^2+^_0_, (2) NM + As_0_ + Ca^2+^_20_, (3) NM + As_100_ + Ca^2+^_0_, (4) NM + As_100_ + Ca^2+^_20_, (5) AM + As_0_ + Ca^2+^_0_, (6) AM + As_0_ + Ca^2+^_20_, (7) AM + As_100_ + Ca^2+^_0_, (8) AM + As_100_ + Ca^2+^_20_. Each treatment had 3 replicates for a total of 24 pots. Two-way and three-way analysis of variance (ANOVA) were used to test experimental data by the statistical software package SPSS 16.0 (SPSS Inc., Chicago, IL, USA). Data were presented as the mean ± SD (n = 3). Significant differences among treatments were determined using one-way analysis of variance (ANOVA) and Tukey’s test (*p* < 0.05).

The AMF strain *Funneliformis mosseae* BGC XZ02A was obtained from the Beijing Academy of Agriculture and Forestry Sciences in Beijing, China. *F. mosseae* was cultured in a sterilized mixture of sand and vermiculite for 12 weeks using sorghum as the host plant. The fresh AMF inoculum was sealed and stored at 4 °C in dark conditions to maintain biological activity. Prior to the experiment, the inoculum exhibited stable infectivity potential, with abundant spores and viable hyphae. This guaranteed reliable mycorrhizal colonization and consistent inoculation efficiency during the whole experiment. The mycorrhizal inoculum was composed of sandy soil, spores (with an approximate concentration of 45 g per dry soil), extraradical hyphae, and colonized root segments of sorghum. For AMF-inoculated pots, 30 g of AMF inoculum was placed 5 cm below the cotton seeds. For the non-AMF inoculated treatment, to ensure a comparable microbial community while excluding AMF, each pot received 50 mL of a microbial suspension. This suspension was prepared by filtering 30 g of unsterilized *F. mosseae* inoculum through a 10 µm ultrafiltration membrane.

The culture medium consisted of soil with a particle size of less than 2 mm, collected from the topsoil layer (5–20 cm depth) in an uncultivated field at Henan University of Science and Technology (Luoyang, China). Sand (<2 mm) was also obtained and thoroughly rinsed with tap water. The soil, sand, and organic matter were then mixed at a volume ratio of 3:1:1, and the resulting mixture was sterilized by autoclaving at 0.11 MPa and 121 °C for 2 h. The physicochemical characteristics of the soil mixture were as follows: 50.52 g/kg organic matter, 83.35 mg/kg available potassium, 40.52 mg/kg available nitrogen, 8.17 mg/kg Olsen phosphorus, and pH 7.8 (soil-water ratio of 1:5).

Cotton seeds of the DaLing cotton 69 variety were procured from the Cotton Research Institute of the Chinese Academy of Agricultural Sciences, Anyang, China. To ensure sterility, these cotton seeds were first sterilized by immersion in 75% ethanol for 20 min, then rinsed thoroughly with deionized water three times. Subsequently, the seeds were induced to germinate in moist, sterilized sand at a consistent temperature of 25 °C for 10 days. From the germinated seeds, healthy seedlings were carefully selected and transplanted into plastic pots measuring 15 cm in diameter and 15 cm in depth. The container was filled with a 2 kg mixture of soil.

To ensure uniform distribution of As throughout the soil under As stress, a solution of Na_3_AsO_4_·12H_2_O was applied to achieve a final As^5+^ concentration of 100 mg·kg^−1^ (based on dry soil weight) and thoroughly mixed with the soil samples. Pots were placed in a temperature-controlled greenhouse with temperatures maintained at 20–35 °C, a 14/10 h light/dark photoperiod, a photosynthetic photon flux density of 800 µmol·m^−2^·s^−1^, and a relative humidity of 60–85%. The soil moisture content was maintained at 75% by regular weight measurements. Additionally, each pot was supplied with 100 mL of modified Hoagland nutrient solution weekly, and pots under the Ca^2+^ supplementation treatment received an additional 20 mmol·L^−1^ CaCl_2_ in the Hoagland nutrient solution [22]. After 60 days of cultivation, the Ca^2+^ supplementation group was subjected to foliar application of 20 mmol·L^−1^ CaCl_2_ solution daily after 18:00. The spray treatment was applied once every 3 days, for a total of 4 applications. Calcium and arsenic were applied before planting, after three months of growth, shoots and roots of AMF-inoculated (AM) and non-inoculated (NM) plants were harvested for subsequent analyses.

### 2.2. Mycorrhizal Colonization Rate

To determine mycorrhizal colonization rate, cotton fine roots were randomly sampled, rinsed thoroughly to remove adhering soil, and cut into 1 cm segments. Then, root segments were placed in a Petri dish and cleared by incubation in 10% (*w*/*v*) KOH solution at 90 °C for 30 min. The stained method of Phillips and Hayman (1970) was modified. After removal of the KOH solution, root segments were rinsed thoroughly with distilled water and acidified in 2% HCl for 5 min at room temperature, and then were stained with 0.05% trypan blue in a lactic acid-glycerol mixture at 90 °C for 20 min, followed by destaining in a lactic acid-glycerol solution for 72 h. Mycorrhizal fungal colonization was assessed under an optical microscope (BX51, Olympus, Tokyo, Japan) according to the method described by Phillips and Hayman (1970). AMF colonization rate was determined using the gridline intersect method, with eight biological replicates per treatment to ensure accuracy and reliability [23].

### 2.3. Plant Measurement and Root Morphology

Harvesting cotton seedlings during the three-month growth period after transplantation, they were thoroughly rinsed with water, and plant height was determined using a graduated ruler. The shoots and roots were separated and dried for at least 48 h at 70 °C to a constant weight to measure root and shoot dry weight.

The root morphology of cotton was evaluated by digital scanning using an Epson Expression 12000 XL root scanner (Seiko Epson Co., Ltd., Tokyo, Japan). The resulting data were subsequently processed using the WinRHIZO root analysis system (Regent Instruments, Sainte Foy, QC, Canada). During measurement, roots were sampled from each treatment and gently rinsed with tap water to remove adhering soil, taking care to avoid damaging fine root structures. Then, the root systems were flattened to reduce root overlap as much as possible.

### 2.4. Gas Exchange and Chlorophyll Fluorescence

Gas exchange parameters, including net photosynthetic rate (*Pn*), stomatal conductance (*gs*), intercellular CO_2_ concentration (*Ci*), and transpiration rate (E), were measured using a Li-Cor 6400 portable photosynthesis measuring system (Li-Cor Inc., Lincoln, NE, USA). The fourth and fifth fully expanded leaves of six randomly selected seedlings were measured between 9:30 and 11:30 AM. The experimental conditions were set as follows: 1500 μmol·m^−2^·s^−1^ (photons) light intensity, 1.5 ± 0.5 kPa leaf-air vapor pressure deficit, 0.5 dm^3^ min^−1^ air flow rate, 350 μmol·mol^−1^ CO_2_ concentration, and 25.0 °C temperature.

Chlorophyll fluorescence parameters were determined using a PAM Chlorophyll Fluorometer (MINI-PAM-II, Heinz Walz GmbH, Germany) between 9:30 and 11:30 AM at room temperature (25.0 °C). Five fully expanded leaves were randomly selected and clamped into the leaf clip chamber, and data were recorded. Measurements for the minimum fluorescence (F0) and maximal fluorescence (Fm) yields were measured in leaves after 20 min of dark adaptation. The steady-state (Fs) and maximal (Fm) fluorescence were determined under light-adapted conditions. The maximum fluorescence yield (Fm) was obtained using a 2.5 s saturating pulse at 1800 μmol·m^−2^·s^−1^, the light-adapted state (F0) was determined following a 2.5 s far-red light at 5 μmmol·m^−2^·s^−1^. The maximum quantum yield of *PSII* (*Fv*/*Fm*, Fv = Fm − F0), the actual quantum yield of PSII electron transport (*ΦPSII* = (Fm − Fs)/Fm), the non-photochemical fluorescence quenching (NPQ = (Fm − Fm)/Fm) and the photochemical fluorescence quenching (qP = (Fm − Fs)/(Fm − Fo) were calculated according to Maxwell and Johnson (2000).

### 2.5. Leaf Stomatal Characteristics

The leaves were collected from the third and fourth fully developed leaves of the tip of cotton seedlings at the end of the experiment. For each treatment, artificial replicas were created by applying polish to the abaxial surface of five leaves, and then used to measure stomatal length, width, and density (number·mm^−2^) and pore aperture. The transparent impressions were secured onto slides, and the central section of each impression was examined under a microscope equipped with a phase-contrast system [24].

### 2.6. Content Determination of Soluble Sugar, Soluble Protein, and Proline

Soluble sugar content was determined following the method described by Li et al. (2000). The reaction mixture was prepared by sequentially adding an appropriate volume of sample extract, 1 mL of 9% phenol solution, and then rapidly adding 5 mL of concentrated sulfuric acid. The mixture was thoroughly vortexed, incubated at room temperature for 30 min, and the absorbance was measured at 485 nm using a UV-visible spectrophotometer (752, Shanghai Precision Instrument & Meter Co., Ltd., Shanghai, China). For protein quantification, 50 μL of ethanolic extract was mixed with 2.5 mL of Bradford’s reagent [25], and the absorbance of the resulting mixture was determined at 595 nm by a UV-visible spectrophotometer, according to the protocol established by Bradford (1976) [25]. To determine the proline content, 1 g of homogenized fresh cotton leaf and root tissue was weighed and extracted in 5 mL of 3% sulfosalicylic acid solution by heating at 100 °C for 20 min. Subsequently, 2 mL of proline extract was mixed with 2 mL acetic acid, and 2 mL acid ninhydrin reagent, and the mixture was incubated at 100 °C for 30 min. After cooling the reaction mixture to room temperature, 4 mL of toluene was added, followed by vortex oscillation for 1 min to ensure thorough mixing. The absorbance of the upper organic phase was measured at a wavelength of 520 nm using a UV-visible spectrophotometer.

### 2.7. Analysis of Antioxidant Enzyme Activity

Antioxidant enzymes were extracted under ice-cold conditions. 0.5 g of fresh leaves was homogenized in 1 mL 0.05 mol/L cold phosphate buffer (pH 7.8), and the homogenate was made up to 5 mL with the same buffer. The homogenate was then centrifuged at 10,000× *g* for 20 min, and the resulting supernatant was used for subsequent enzyme activity assays. Superoxide dismutase (SOD, EC 1.15.1.1) activity was determined spectrophotometrically at 560 nm using a reaction system containing phosphate buffer, methionine, nitroblue tetrazolium (NBT), EDTA-Na_2_, and riboflavin. The reaction was carried out under 4000 lx light for 20 min with a dark control, and one unit (U) of SOD activity was defined as the amount of enzyme required to inhibit the photochemical reduction in NBT by 50%. Ref. [24] CAT (EC 1.11.1.6) activity was assayed by the potassium permanganate titration method: the reaction mixture was incubated at 30 °C for 10 min, then acidified with 10% H_2_SO_4_, and the remaining H_2_O_2_ was titrated with 0.1 mol/L KMnO_4_. The CAT activity was expressed as milligrams of H_2_O_2_ decomposed per gram of fresh weight per minute [25]. Peroxidase (POD, EC 1.11.1.7) activity was quantified spectrophotometrically at 470 nm, with one unit (U) defined as the amount of enzyme that catalyzes the formation of 1 g of tetraguaiacol per min [26].

### 2.8. Content Determination of Malondialdehyde

Fresh leaf and root tissues were sampled and dried with filter paper. A total of 0.5 g of each tissue was homogenized in pre-cooled 10% trichloroacetic acid (TCA) on ice. The homogenate was centrifuged at 4000 r/min for 15 min at 4 °C. Afterward, 2 mL of supernatant was mixed with 2 mL of 0.6% thiobarbituric acid (TBA) solution. The mixture was heated in a boiling water bath for 15 min, cooled naturally and centrifuged at 3000 r/min for 10 min. The absorbance values at 450 nm, 532 nm and 600 nm were determined by spectrophotometry. The malondialdehyde (MDA) concentration and content were calculated according to the corrected absorbance formula, and the final result was expressed as μmol/g fresh weight [25].

### 2.9. Determination of K^+^ and Ca^2+^ Content

The dried cotton leaves and roots were ground into fine powder. One gram samples were weighed into 25 mL porcelain crucibles and gradually heated on an electric furnace until complete carbonization was achieved (no white smoke emission). The samples were then transferred to a muffle furnace and ashed at 500 °C for 4–6 h until white or light gray ash was obtained. After cooling, 10 mL of 6 mol/L HCl was added to dissolve the ash, followed by filtration into 50 mL volumetric flasks. The residues were repeatedly washed with 2% HCl until reaching the final volume. The filtrate was diluted as needed for analysis (sample test solution), with a reagent blank prepared simultaneously. The K^+^ and Ca^2+^ contents in cotton leaves and roots were determined using atomic absorption spectrophotometry (Persee TAS-990AFG, Beijing, China).

### 2.10. Statistical Analysis

To investigate the multivariate relationships among growth parameters, antioxidant activity, and further pinpoint the dominant factors shaping sample distribution in cotton roots and leaves, principal component analysis (PCA) was performed with Origin 2023 software (OriginLab Inc., Northampton, MA, USA). Additionally, the Mantel test was carried out using the linkET, dplyr, and ggplot2 packages in R4.5.2 to verify the statistical significance of correlations between physiological-metabolic traits and the growth performance of cotton shoots and roots.

## 3. Results

### 3.1. Mycorrhizal Colonization Rate

**Figure 1:**
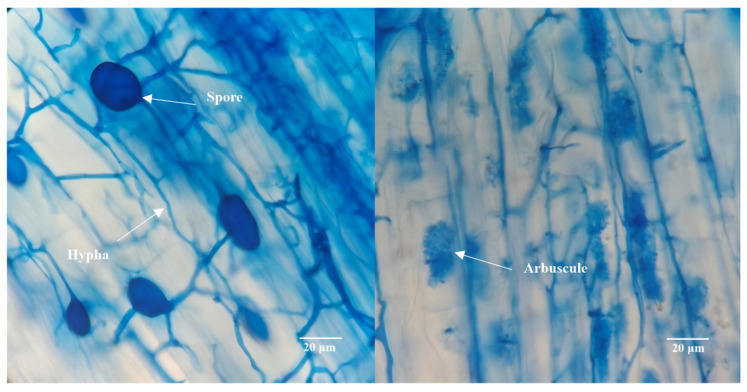
Trypan blue staining of cotton roots colonized by *F. mosseae*.

**Figure 2:**
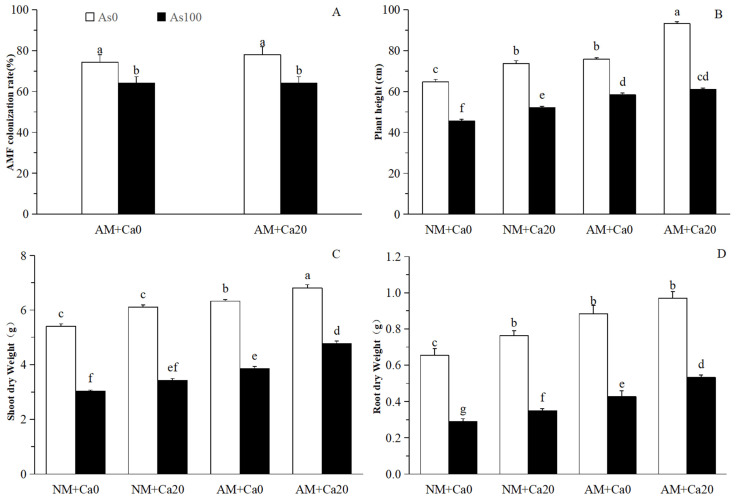
Effects of exogenous calcium and arsenic stress on AMF colonization rate and growth parameters of cotton seedlings. (**A**) AMF colonization rate (%); (**B**) Plant height (cm); (**C**) Shoot dry weight (g); (**D**) Root dry weight (g). Note: Different letters indicate a significant difference at *p* ≤ 0.05. Abbreviations: As_0_: Non-As stress; As_100_: 100 mg/kg As stress; NM: non-mycorrhizal inoculation; AM: *F. mosseae* inoculation; Ca_0_: without exogenous Ca^2+^ application; Ca_20_: 20 mmol·L^−1^ exogenous Ca^2+^ application.

Microscopic examination revealed that the AMF species *F. mosseae* successfully established colonization within cotton roots (Figure 1). Cotton seedlings subjected to *F. mosseae* inoculation developed typical arbuscules, hyphae and vesicular structures in the root cortex. Still, these mycorrhizal structures were absent in non-inoculated cotton. The AMF colonization rates in cotton roots exhibited no significant difference between Non-Ca^2+^ (74.35% under Non-As stress and 64.03% under As stress) and Ca^2+^ application (78.02% under Non-As stress and 64.03%) under the same As stress conditions (Figure 2A). Nevertheless, the AMF colonization rates were notably higher in both Non-Ca^2+^ and Ca^2+^ application treatments grown under Non-As stress conditions, compared with those cultivated under As stress conditions.

### 3.2. Growth Parameters and Root Morphology

**Figure 3:**
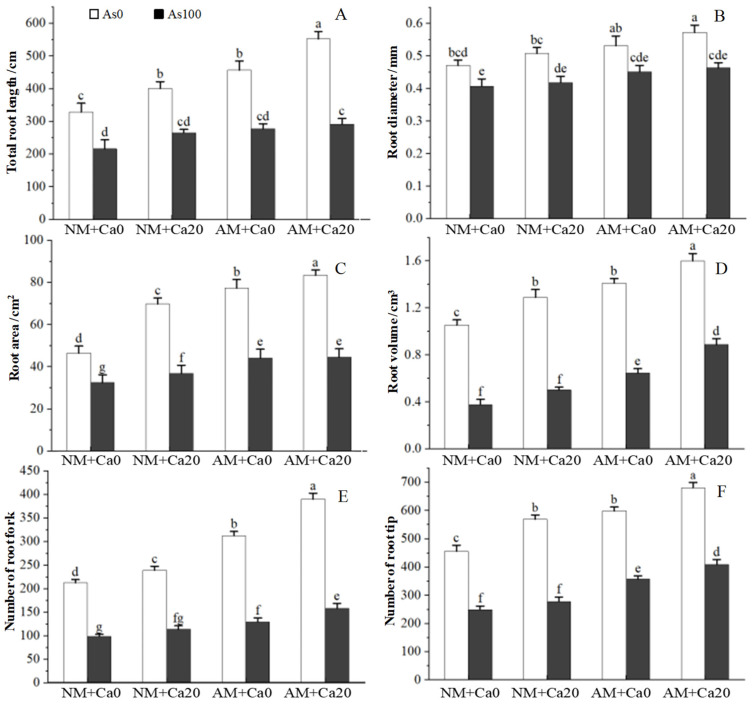
Effects of AMF and exogenous calcium on root morphological parameters of cotton seedlings under arsenic stress. (**A**) Total root length (cm); (**B**) Root diameter (mm); (**C**) Root area (cm^2^); (**D**) Root volume (cm^3^); (**E**) Number of root forks; (**F**) Number of root tips. Note: Different letters indicate a significant difference at *p* ≤ 0.05. Abbreviations: As_0_: Non-As stress; As_100_: 100 mg/kg As stress; NM: non-mycorrhizal inoculation; AM: *F. mosseae* inoculation; Ca_0_: without exogenous Ca^2+^ application; Ca_20_: 20 mmol·L^−1^ exogenous Ca^2+^ application.

Non-Ca^2+^ and Ca^2+^ applied cotton seedlings subjected to As stress showed declines in plant height, shoot dry weight and root dry weight, compared with those under Non-As stress condition (Figure 2). Both Ca^2+^ application and AMF inoculation exerted positive effects on these growth parameters. Specifically, under As stress, AMF inoculation increased plant height (by 28.05% and 15.18%) (Figure 2B), shoot dry weight (by 26.64% and 38.78%) (Figure 2C), and root dry weight (by 53.57% and 51.43%) in Non-Ca^2+^ and Ca^2+^ applied treatments (Figure 2D), respectively. Similarly, under As stress, Ca^2+^ application enhanced plant height (by 14.20% and 4.43%) (Figure 2B), shoot dry weight (by 12.83% and 23.64%) (Figure 2C), and root dry weight (by 20.69% and 23.26%) in non-mycorrhizal (NM) and mycorrhizal (AM) cotton seedlings (Figure 2D), respectively. Two-way ANOVAs revealed that all root morphological parameters were significantly affected by As stress, AMF inoculation, and Ca^2+^ application (Figure 3). Total root length, root area, and number of root forks had significant interactive effects between As stress and AMF inoculation, and between As stress and Ca^2+^ application. Root area also showed a significant three-way interaction, while root diameter, root volume, and number of root tips did not (Figure 3 and Table 1).

### 3.3. Gas Exchange

The results revealed that As stress, AMF inoculation, and Ca^2+^ application influenced the parameters of gas exchange in cotton leaves, including net photosynthetic rate (*Pn*), stomatal conductance (*gs*), transpiration rate (*E*), and intercellular CO_2_ concentration (*Ci*) in cotton seedlings (Table 2). Irrespective of AMF inoculation and Ca^2+^ application, cotton seedlings exposed to As stress generally exhibited lower values of *Pn*, *gs*, *E*, and *Ci*, compared to those under the Non-As stress condition. Under As stress, AMF inoculated seedlings typically showed higher *Pn* values than non-mycorrhizal seedlings in both Non-Ca^2+^ and Ca^2+^ application treatments. Exogenous Ca^2+^ application also had a positive effect on the parameters of gas exchange in cotton seedlings. Generally, Ca^2+^ application led to increased values of *Pn*, *gs*, *E*, and *Ci*, compared to the Non-Ca^2+^ application treatment under As stress for both non-mycorrhizal (NM) and mycorrhizal (AM) seedlings.

The three-factor analysis further elucidated that *Pn*, *gs*, *E*, and *Ci* were all significantly affected by As stress, AMF inoculation, and Ca^2+^ application (Table 1). By the method of Two-way ANOVAs, the interaction between As stress and AMF inoculation had a significant impact on *Pn*, and the interaction between AMF and Ca^2+^ application had a significant effect on *Ci*. Moreover, the three-way ANOVAs indicated that *Pn* and *Ci* were significantly affected by As×AMF×Ca^2+^.

### 3.4. Chlorophyll Fluorescence

As stress significantly reduced the effective quantum yield of PSII (*ΦPSII*), maximum quantum yield of PSII (*Fv/Fm*), and photochemical quenching coefficient (*qP*), while increasing non-photochemical quenching coefficient (NPQ) (Table 3). Under As stress, AMF inoculation increased *ΦPSII* by 43.92% and 20.09%, *Fv/Fm* by 8.32% and 9.31%, and *qP* by 9.65% and 7.72%, while decreasing *NPQ* by 6.26% and 10.14% in Non-Ca^2+^ and Ca^2+^ application treatments, respectively, compared to non-AMF inoculated plants. With Ca^2+^ application under As stress, *ΦPSII* increased by 30.21% and 8.65%, *Fv/Fm* by 3.68% and 4.63%, and *qP* by 5.42% and 3.57%, while *NPQ* decreased by 2.98% and 7.00% in non-AMF and AMF inoculation treatments, respectively, compared to Non-Ca^2+^ cotton seedlings. Two-way ANOVAs revealed that *ΦPSII*, *Fv/Fm*, and *qP* were significantly affected by the interactions of As×AMF, As×Ca^2+^, and, notably, Fv/Fm was significantly influenced by As×AMF (Table 1). Three-way ANOVAs indicated that *ΦPSII* was significantly affected by the interaction of As×AMF×Ca^2+^, while the other parameters (*Fv/Fm*, *qP*, and *NPQ*) did not show significant responses to this three-way interaction (Table 1).

### 3.5. Stomatal Structure and Characteristics

**Figure 4:**
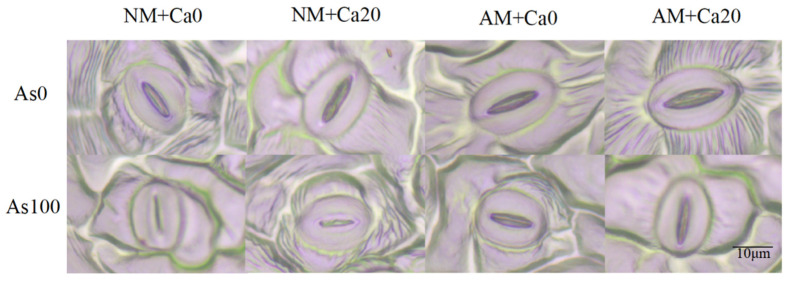
Effect of AMF and exogenous calcium on stomatal morphology on cotton leaves under arsenic stress. Abbreviations: As_0_: Non-As stress; As_100_: 100 mg/kg As stress; NM: non-mycorrhizal inoculation; AM: *F. mosseae* inoculation; Ca_0_: without exogenous Ca^2+^ application; Ca_20_: 20 mmol·L^−1^ exogenous Ca^2+^ application.

AMF inoculation and Ca^2+^ application obviously affected the stomatal structure and characteristics of cotton leaves under As stress (Figure 4 and Table 4). When subjected to As stress alone (As100 + NM + Ca0), the stomatal morphology of cotton leaves exhibited a more closed state, with reduced stomatal length (15.49 ± 0.24 μm), width (11.57 ± 0.37 μm), and pore aperture (2.63 ± 0.11 μm) compared to the control (As0 + NM + Ca0).

Both AMF inoculation and Ca^2+^ application alone alleviated the inhibitory effect of As stress on stomatal structure and characteristics of cotton leaves, and their combined application showed a better regulatory effect. Under As stress, compared with the non-inoculated and non-Ca^2+^ application treatment (As_100_ + NM + Ca_0_), the stomatal length, width, pore aperture, and density of the AMF-inoculated treatment (As_100_ + AM + Ca_0_) increased by 6.97%, 9.51%, 19.77%, and 21.92% respectively, while those of the non-inoculated but Ca^2+^ application treatment (As_100_ + NM + Ca_20_) increased by 5.62%, 7.09%, 15.59%, and 14.24% respectively (Table 4). The combined application of AMF and Ca^2+^ (As_100_ + AM + Ca_20_) further improved the stomatal characteristics, with stomatal length, width, pore aperture, and density reaching 16.91 μm, 12.88 μm, 3.32 μm, and 313.67 number·mm^−2^, respectively, which were the highest among all As stress treatment groups (Table 4).

Statistical analysis showed that As stress, AMF inoculation, and exogenous Ca^2+^ application all had extremely significant effects on stomatal length, width, pore aperture, and density (Table 1). The interaction between As and AMF had a significant effect on stomatal length, and the interaction between As and Ca^2+^ had no significant effect on all stomatal characteristics. The interaction between AMF and Ca^2+^ had no significant effect on all indicators, while the three-way interaction of As×AMF×Ca^2+^ only had a significant effect on pore aperture (Table 1).

### 3.6. Osmotic Regulating Substance

As stress significantly reduced the contents of soluble sugar, soluble protein, and proline in cotton leaves and roots. Under As stress, AMF-inoculated seedlings showed 80.00%/51.55% (Non-Ca^2+^/Ca^2+^ application) higher soluble sugar content, 23.06%/23.15% higher soluble protein content, and 17.66%/10.52% higher proline content in leaves, as well as 50.00%/13.70% higher soluble sugar content, 131.66%/20.12% higher soluble protein content, and 20.95%/14.38% higher proline content in roots, compared to Non-inoculated seedlings (Table 5).

With Ca^2+^ application under As stress, soluble sugar content increased by 56.21%/31.51% (NM/AM), soluble protein by 17.75%/17.84%, and proline by 13.29%/6.42% in leaves, as well as in roots. Soluble sugar increased by 47.50%/8.20%, soluble protein by 111.91%/9.88%, and proline by 15.11%/8.86%, compared to Non-Ca^2+^ applied seedlings (Table 5). Even under Non-As stress, AMF inoculation and Ca^2+^ application still promoted osmolyte accumulation, though the promotion effect was weaker than that under As stress.

Three-way ANOVAs revealed that soluble sugar, soluble protein, and proline contents in both tissues were significantly affected by As stress, AMF, and Ca^2+^ (*p* < 0.001). For soluble sugar: leaf content was affected by As×Ca^2+^ interaction, root content by As×AMF and AMF×Ca^2+^ interactions (Table 1). Leaf content of soluble protein was affected by all pairwise interactions and the three-way interaction (As×AMF×Ca^2+^), while root content of a soluble protein was only affected by the AMF×Ca^2+^ interaction. Leaf content of proline was affected by As×AMF and As×Ca^2+^ interactions, root content of proline by AMF×Ca^2+^ interaction; the three-way interaction had no significant effect on any osmolyte content (Table 1).

### 3.7. Antioxidant Enzyme Activities

**Figure 5:**
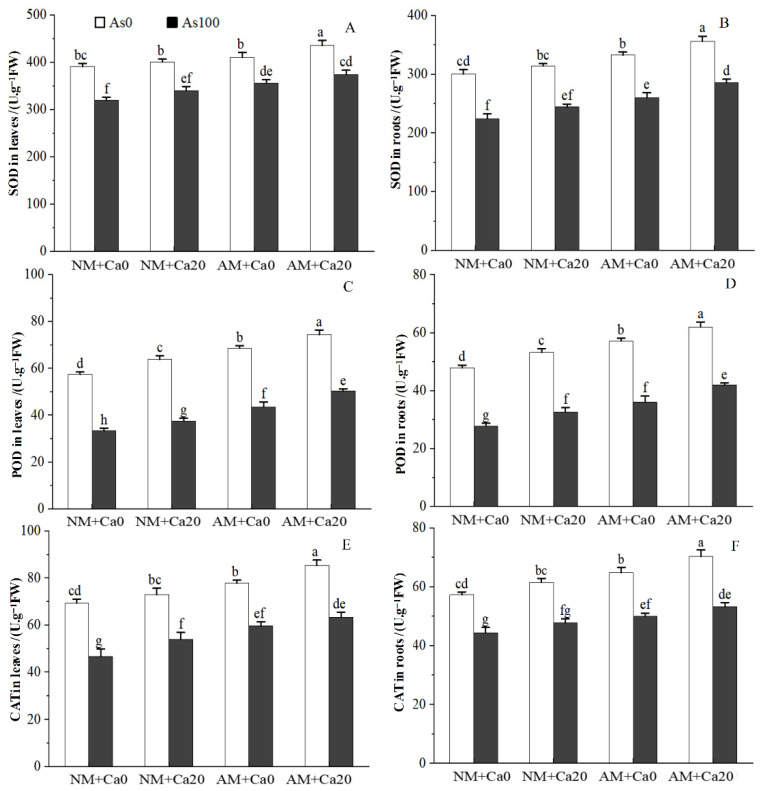
Effects of AMF and exogenous calcium on antioxidant enzyme activities in leaves and roots of cotton seedlings under arsenic stress. (**A**) SOD activity in leaves (U·g^−1^FW); (**B**) SOD activity in roots (U·g^−1^FW); (**C**) POD activity in leaves (U·g^−1^FW); (**D**) POD activity in roots (U·g^−1^FW); (**E**) CAT activity in leaves (U·g^−1^FW); (**F**) CAT activity in roots (U·g^−1^FW). Note: Different letters indicate a significant difference at *p* ≤ 0.05. Abbreviations: As_0_: Non-As stress; As_100_: 100 mg/kg As stress; NM: non-mycorrhizal inoculation; AM: *F*. *mosseae* inoculation; Ca_0_: without exogenous Ca^2+^ application; Ca_20_: 20 mmol·L^−1^ exogenous Ca^2+^ application.

As stress significantly inhibited the activities of antioxidant enzymes (superoxide dismutase (SOD), peroxidase (POD), and catalase (CAT)) in cotton leaves and roots, while AMF inoculation and Ca^2+^ application effectively alleviated this inhibitory effect by enhancing these enzyme activities (Figure 5). Under As stress, AMF-inoculated (AM) seedlings showed higher antioxidant enzyme activities than Non-inoculated (NM) seedlings. In leaves, AMF inoculation increased SOD, POD, and CAT activities by 11.37%/9.88% (Non-Ca^2+^/Ca^2+^) (Figure 5A), 30.66%/34.01% (Figure 5C), and 27.84%/17.21% (Figure 5E), respectively. In roots, the corresponding increases were 16.01%/16.98% (Figure 5B), 29.45%/28.50% (Figure 5D), and 12.81%/11.54% (Figure 5F). Ca^2+^ application also promoted enzyme activities under As stress: in NM seedlings, Ca^2+^ application increased SOD, POD, and CAT activities in leaves by 6.37%, 12.57%, and 15.81% (Figure 5A,C,E), and activities in roots by 8.83%, 17.35%, and 7.54% (Figure 5B,D,F). In AMF-inoculated seedlings, the increases were 4.95%, 15.47%, 6.17%in leaves (Figure 5A,C,E) and 9.73%, 16.48%, 6.32% in roots (Figure 5B,D,F), compared to Non-Ca^2+^ treatments.

Three-way ANOVAs (Table 1) revealed that SOD, POD, and CAT activities in both leaves and roots were significantly affected by As stress, AMF inoculation, and Ca^2+^ application (Table 1). However, no significant interactions were detected among As×AMF, As×Ca^2+^, AMF×Ca^2+^, or the three-way interaction (As×AMF×Ca^2+^) for any antioxidant enzyme activity in leaves or roots (all NS), indicating that AMF and exogenous calcium independently enhance cotton’s antioxidant defense system under As stress (Table 1).

### 3.8. Content of Malondialdehyde

**Figure 6:**
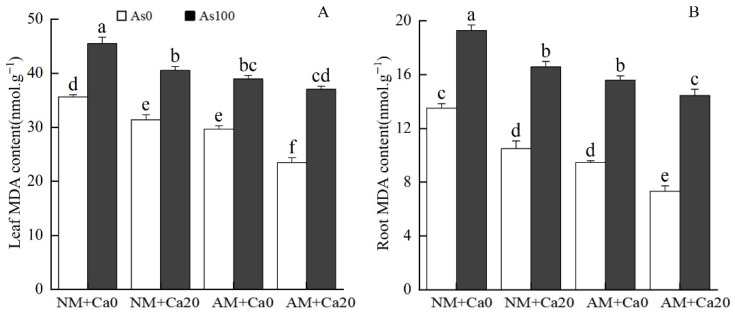
Effects of AMF and exogenous calcium on malondialdehyde content in leaves and roots of cotton seedlings under arsenic stress. (**A**) MDA content in leaves; (**B**) MDA content in roots. Note: Different letters indicate a significant difference at *p* ≤ 0.05. Abbreviations: As_0_: Non-As stress; As_100_: 100 mg/kg As stress; NM: non-mycorrhizal inoculation; AM: *F*. *mosseae* inoculation; Ca_0_: without exogenous Ca^2+^ application; Ca_20_: 20 mmol·L^−1^ exogenous Ca^2+^ application.

Under arsenic stress, AMF inoculation reduced leaf MDA content by 14.42% and 8.57%, and root MDA content by 19.11% and 12.87% in non-calcium and calcium treatments, respectively, compared to non-inoculated plants (Figure 6). With exogenous calcium application under arsenic stress, leaf MDA content decreased by 10.94% and 4.85%, and root MDA content decreased by 13.99% and 7.35% in non-AMF and AMF treatments, respectively, relative to non-calcium plants.

Three-way ANOVA revealed significant main effects of AMF, Ca^2+^, and arsenic stress on MDA content in both leaves and roots. Significant AMF×Ca^2+^ interactions were observed for leaf MDA content, while the three-factor interaction significantly affected root MDA content rather than leaf MDA content (Table 1). Collectively, AMF and exogenous calcium could jointly alleviate arsenic-induced membrane lipid peroxidation by improving antioxidant enzyme activities and reducing MDA accumulation, thereby maintaining cell membrane stability under As stress.

### 3.9. Ca^2+^ and K^+^ Contents

**Figure 7:**
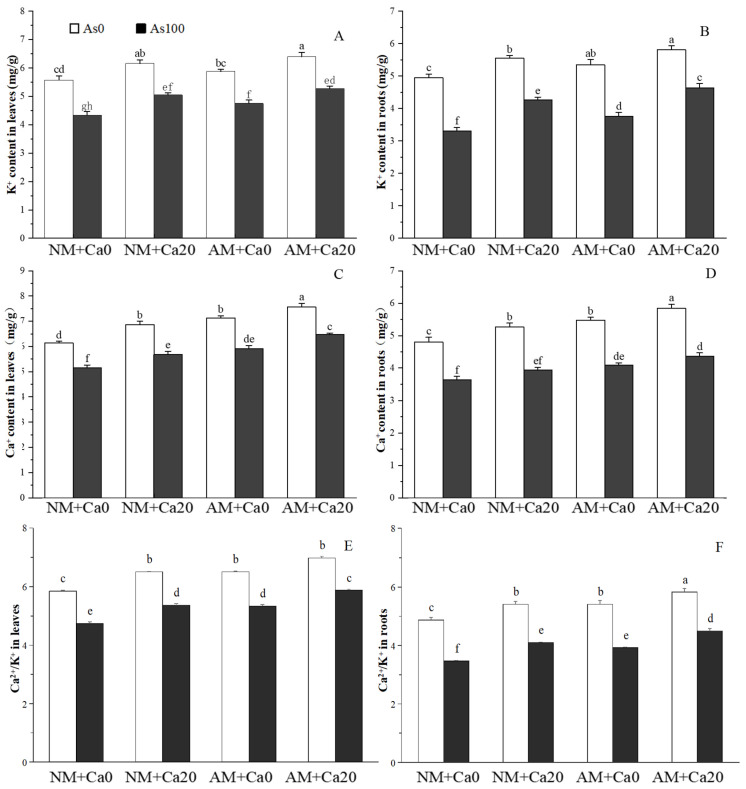
Effects of AMF and exogenous calcium on K^+^ and Ca^2+^ contents in leaves and roots of cotton seedlings under arsenic stress. (**A**) K^+^ content in leaves; (**B**) K^+^ content in roots; (**C**) Ca^2+^ content in leaves; (**D**) Ca^2+^ content in roots; (**E**) Ca^2+^/K^+^ ratio in leaves; (**F**) Ca^2+^/K^+^ ratio in roots. Note: Different letters indicate a significant difference at *p* ≤ 0.05. Abbreviations: As_0_: Non-As stress; As_100_: 100 mg/kg As stress; NM: non-mycorrhizal inoculation; AM: *F*. *mosseae* inoculation; Ca_0_: without exogenous Ca^2+^ application; Ca_20_: 20 mmol·L^−1^ exogenous Ca^2+^ application.

As stress significantly induced a sharp decline in K^+^ and Ca^2+^ contents, and disrupted the Ca^2+^/K^+^ balance in cotton leaves and roots (Figure 7), while AMF inoculation and Ca^2+^ application effectively mitigated this inhibitory effect and regulated the Ca^2+^/K^+^ balance. Under As stress, AMF-inoculated (AM) seedlings exhibited significantly higher K^+^ and Ca^2+^ contents than non-inoculated (NM) seedlings. In leaves, AMF inoculation increased K^+^ content by 9.45% (Non-Ca^2+^) and 4.55% (Ca^2+^) (Figure 7A), and Ca^2+^ content by 14.96% and 13.97%, respectively (Figure 7C). In roots, the corresponding increases were 13.71% and 8.69% for K^+^ (Figure 7B), and 12.36% and 10.57% for Ca^2+^ (Figure 7D). Ca^2+^ application also enhanced K^+^ and Ca^2+^ accumulation under As stress: in NM seedlings, Ca^2+^ application elevated leaf K^+^ and Ca^2+^ contents by 16.36% and 10.36% (Figure 7A,C), and root contents by 28.83% and 8.33% (Figure 7B,D); in AMF-inoculated seedlings, the increases were 11.16% and 9.41% in leaves (Figure 7A,C) and 23.14% and 6.60% in roots (Figure 7B,D) compared to Non-Ca^2+^ treatments. The Ca^2+^/K^+^ ratio in leaves was consistently higher than that in roots across all treatments, and both AMF and Ca^2+^ application restored this ratio closer to Non-As stress levels (Figure 7E,F).

Three-way ANOVAs showed that the K^+^ content in roots was significantly affected by the interaction of As×Ca^2+^, and the Ca^2+^ content in roots was significantly influenced by the interaction of As×AMF. The K^+^ content in leaves, Ca^2+^ content in leaves, and Ca^2+^/K^+^ ratio in both leaves and roots were not affected by any pairwise interactions (As×AMF, As×Ca^2+^, or AMF×Ca^2+^) (Table 1). All of the above parameters (K^+^ content, Ca^2+^ content, and Ca^2+^/K^+^ ratio in leaves and roots) were significantly affected by As stress, AMF inoculation, and exogenous Ca^2+^ application (all *p* < 0.001) (Table 1).

### 3.10. Interactions of Antioxidant Activity, Osmotic Regulation, and Ion Balance in Cotton

**Figure 8:**
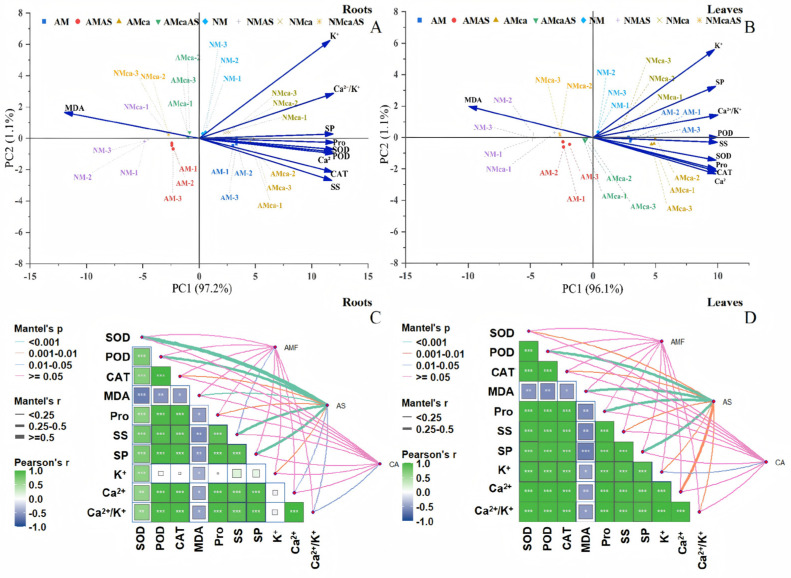
The interrelationships among antioxidant activity, osmotic regulation, and ion balance following AMF inoculation, Ca^2+^ application and As stress were elucidated by principal component analysis (PCA) (**A**,**B**), identifying the relative contributions of antioxidant activity, osmotic regulation, and ion balance to intertreatment differences, mantel tests (**C**,**D**) uncovering the relationship between antioxidant activity, osmotic regulation, and ion balance. Note: Different letters indicate significant differences at *p* ≤ 0.05. Significance levels are denoted as: NS, not significant; * *p* < 0.05; ** *p* < 0.01; *** *p* < 0.001. Abbreviations: As: As stress; NM: non-mycorrhizal inoculation; AM: *F. mosseae* inoculation; Ca: exogenous Ca^2+^ application; SOD: superoxide dismutase; POD: peroxidase; CAT: catalase; MDA: malondialdehyde; Pro: proline; SS: soluble sugars; SP: soluble protein.

A principal component analysis (PCA) was conducted to evaluate the impacts of AMF inoculation, Ca^2+^ application and As stress on antioxidant parameters, osmolytes, and ion balance in cotton. The PCA score plot explained 97.2% and 96.1% of the total variation in roots and leaves, respectively (Figure 8A,B). The samples subjected to distinct AMF inoculation, Ca^2+^ application and As stress showed robust separation (Figure 8A). Moreover, MDA were positively associated with Ca^2+^ application, and Pro (proline), Ca^2+^, SOD, POD, CAT, and SS (soluble sugar) were clustered with AMF inoculation in roots under Ca^2+^ application conditions (Figure 8A). Pro clustered with SOD, POD, Ca^2+^, CAT, SS, Ca^2+^/K^+^, SP (soluble protein) and K^+^ in leaves. Furthermore, Ca^2+^/K^+^, SP, POD and K^+^ were clustered around AMF inoculation, and SS, POD, Pro, CAT, and Ca^2+^ were associated with AMF inoculation in leaves under Ca^2+^ application conditions (Figure 8B). Spearman’s correlation analysis showed significantly positive correlations between SOD, POD, and CAT. In contrast, MDA showed a significant negative correlation with SOD, POD, CAT, Pro, and the Ca^2+^/K^+^ ratio in roots (Figure 8C). MDA was significantly negatively correlated with SOD, POD, CAT, and the Ca^2+^/K^+^ ratio in leaves. Among them, it had the strongest negative correlation with CAT (Figure 8D). Mantel test results showed significant correlations between different treatment groups (AMF, AS, CA), and the AMF inoculation group was highly positively correlated with SOD, POD, CAT, Pro, and the Ca^2+^/K^+^ ratio in roots; the As stress group was significantly positively correlated with SS and SP in roots. The Ca^2+^ application group was significantly positively correlated with Ca^2+^, K^+^, and the Ca^2+^/K^+^ ratio in roots (Figure 8C). In the leaves, the AMF inoculation group showed a significant positive correlation with SOD, POD, and CAT; the As stress group showed a moderate positive correlation with SS and SP. In contrast, the Ca^2+^ application group showed a significant positive correlation with Ca^2+^ and the Ca^2+^/K^+^ ratio (Figure 8D).

## 4. Discussion

In this study, our core hypothesis was that arbuscular mycorrhizal fungi (AMF) and exogenous calcium would synergistically enhance arsenic tolerance in cotton by improving growth, photosynthesis, antioxidant capacity, osmotic adjustment, and ion homeostasis. This hypothesis was strongly corroborated by the present results. Combined AMF inoculation and Ca^2+^ application exhibited significantly better effects than either treatment alone in alleviating As-induced damage, which confirmed the synergistic mechanism. These findings have important implications for understanding microbe-mineral interactions under heavy metal stress and provide an eco-friendly strategy for safe cotton production and phytoremediation in As-contaminated soils.

### 4.1. Synergistic Promotion of Cotton Growth and Root Morphology by AMF Inoculation and Ca^2+^ Application Under as Stress

As stress directly inhibited cotton growth by disrupting nutrient absorption, damaging cell structures, and inducing oxidative stress [2]. Our results showed that As stress reduced plant height, shoot dry weight, and root dry weight of cotton seedlings (Figure 2), which was consistent with the growth inhibition observed in As-stressed *Robinia pseudoacacia* and *Sophora davidii* [4,14]. This growth inhibition was attributed to multiple interconnected physiological and biochemical disruptions induced by As accumulation [27]. Root morphology, a critical determinant of nutrient and water uptake, was also severely impaired by As stress in this study, as reflected by reduced total root length, root area, and root tip number of cotton (Figure 3). As stress damaged root cell membranes and inhibited cell division, root elongation and branching, which further restricted water and nutrient acquisition in cotton [14,27].

AMF inoculation effectively mitigated As-induced growth inhibition of cotton seedlings, as evidenced by significant increases in plant height, shoot dry weight, and root dry weight (Figure 2), as well as improved root morphological traits in this study, which was also consistent with previous findings in *R. pseudoacacia* and *S. davidii* seedlings [4,14]. The mycorrhizal hyphal network expanded the root absorption range, improving the acquisition of limited nutrients (e.g., P, N, Ca^2+^) under As stress [11]. Moreover, AMF immobilized As ions in fungal hyphae or cell walls, reducing their translocation to aboveground tissues and minimizing cellular toxicity [4,28]. AMF inoculation also protected the root architecture of cotton under As stress by promoting root branching and elongation, which is associated with the regulation of hormone metabolism (e.g., increased GA and IAA levels) and the upregulation of genes involved in root development [22].

In plants, Ca^2+^ ions are primarily taken up from the soil through root tips and subsequently transported to the aerial shoots via the xylem, and soil Ca^2+^ deficiency can adversely impair plant growth and stress resistance [22]. Exogenous Ca^2+^ application also had a positive effect on cotton growth and root development under As stress (Figure 2), which was consistent with the role of Ca^2+^ as a critical nutrient and signaling molecule in plant stress responses. Our results showed that Ca^2+^ application increased root dry weight, total root length, and root area in cotton seedlings under As stress, which further improved the cotton’s ability to absorb water and nutrients (Figure 3). For root growth, Ca^2+^ directly promoted root cell elongation and division by regulating cell wall extensibility and cytoskeletal dynamics, and it also regulated the expression of root development-related genes [22,29].

In this study, the synergistic effect between AMF inoculation and Ca^2+^ application produced a more pronounced promotion of cotton growth and root morphology than either treatment alone, which was attributed to Ca^2+^-enhanced AMF colonization and AMF-facilitated Ca^2+^ uptake and transport (Figure 2). Ca^2+^ application promoted AMF colonization and symbiosis establishment. The effects of the combined treatment with AMF and Ca^2+^ on peanut growth were significantly better than those of single treatments with AMF or Ca^2+^ alone under saline alkali stress [16]. The adequate Ca^2+^ supply enhanced the formation of arbuscules and hyphal networks, improving the efficiency of nutrient exchange between AMF and host plants [22]. In return, AMF hyphal networks increased Ca^2+^ uptake by expanding the root absorption area and by upregulating Ca^2+^ transporters, thereby maintaining optimal Ca^2+^ levels required for stress signaling and membrane stability [22,30]. AMF also activated Ca^2+^-dependent signaling pathways that promote root growth and resistance to As stress in cotton [31]. The combination of AMF and Ca^2+^ had a stronger regulatory effect on the root morphology of cotton, leading to a more robust root system with increased total root length, root area, and root fork number in this study, which improved root architecture, significantly expanded the soil exploration range, facilitating the acquisition of water and nutrients even under As stress [16,22].

### 4.2. Improvement of Photosynthetic Capacity Through Regulation of Stomatal Function and Photosystem Stability

Photosynthesis was highly sensitive to As stress, with inhibition typically resulting from stomatal limitation and non-stomatal damage [27]. Chlorophyll fluorescence, as an indicator of the photochemical efficiency of photosystem II, offered insights into the extent to which As stress impaired the photosynthetic apparatus [32]. In this study, As stress had comprehensive inhibitory effects on the photosynthetic physiology of cotton seedlings by suppressing gas exchange parameters, altering chlorophyll fluorescence traits, and impairing stomatal structure, which indicated that the photosynthetic apparatus had been damaged (Table 2 and Table 3). As ions were transported across the plasma membrane by phosphate transporters and accumulated in chloroplasts, where they disrupted thylakoid membrane integrity, inhibited Calvin cycle enzymes (e.g., Rubisco), and damaged the photosystem II (*PSII*) reaction center, manifested by reduced *ΦPSII*, *Fv/Fm*, and *qP* [4,33]. As stress also disrupted thylakoid membrane structure and inhibited *PSII* core protein synthesis, reducing light energy absorption and conversion efficiency [34].

In this study, AMF inoculation effectively alleviated As-induced photosynthetic inhibition, as evidenced by elevated gas exchange parameters, reversed deterioration of chlorophyll fluorescence traits, and improved stomatal structure, compared with non-mycorrhizal plants (Table 2 and Table 3), and this improvement was closely associated with AMF-mediated optimization of stomatal function. The increase in stomatal length, width, pore aperture, and density in AMF-inoculated cotton promoted CO_2_ uptake and water transpiration (Figure 4 and Table 4), thereby alleviating the stomatal limitation of photosynthesis caused by As stress [4]. AMF symbiosis enhanced the synthesis of auxin (IAA) and reduced the accumulation of abscisic acid (ABA) in host plants, which was conducive to stomatal development and opening [11]. AMF inoculation significantly increased *ΦPSII*, *Fv*/*Fm*, and *qP* while decreasing NPQ in cotton leaves in this study, indicating that AMF can protect *PSII* from As-induced damage (Table 3). AMF inoculation also improved stomatal characteristics (length, width, pore aperture) by enhancing nutrient supply and ROS scavenging, thereby increasing *gs* and *Ci* [14]. It stabilized the photosynthetic apparatus by reducing ROS-induced damage to thylakoid membranes, as indicated by higher Fv/Fm and *ΦPSII* values [35].

In this study, exogenous Ca^2+^ application also exhibited a positive effect on the photosynthetic capacity of cotton seedlings under As stress, and the increase in gas exchange parameters (*Pn*, *gs*, *E*, *Ci*) in Ca^2+^-applied cotton seedlings was mainly due to the improvement of stomatal function (Table 2 and Table 4). Exogenous Ca^2+^ regulated stomatal movement by modulating the cytosolic Ca^2+^ concentration, promoting stomatal opening and CO_2_ diffusion [36]. As a second messenger, Ca^2+^ also upregulated the expression of genes encoding *PSII* core subunits, facilitating photosystem repair and enhancing light energy conversion efficiency [37]. Ca^2+^ participated in the signal transduction pathway of stomatal movement, stabilized the structure of the guard cell membrane and maintained the integrity of stomatal guard cells by cross-linking with cell wall components and regulating ion channel activity, thereby alleviating As-induced stomatal closure [31,38]. In this study, Ca^2+^ application alleviated the excessive closure of stomata caused by As stress, and the increased stomatal length, width, and pore aperture in Ca^2+^-applied cotton seedlings promoted CO_2_ diffusion and improved photosynthetic carbon assimilation efficiency (Table 4).

The combination of AMF inoculation and Ca^2+^ application showed a more significant regulatory effect on photosynthetic capacity than individual AMF inoculation or Ca^2+^ application, which was reflected in the highest values of gas exchange parameters and stomatal characteristics in the As_100_ + AM + Ca_20_ treatment. The synergistic effect of AMF inoculation and Ca^2+^ application on gas exchange was highlighted by the three-way ANOVA result that As×AMF×Ca^2+^ significantly affected *Pn* and *Ci* (Table 1). This synergy may derive from mutual promotion between the two treatments: Ca^2+^ application enhanced AMF colonization and hyphal growth by stabilizing the root rhizosphere environment [22], while AMF expanded the root absorption range to improve Ca^2+^ uptake efficiency [39]. Together, AMF inoculation and Ca^2+^ application comprehensively alleviated As-induced stomatal limitation (via optimizing gs) and non-stomatal limitation (via protecting chloroplast function), leading to the highest *Pn* and *Ci* in the combined treatment group. The two-way ANOVA results showed that the interaction between As stress and Ca^2+^ application significantly affected *ΦPSII* and *Fv/Fm*, indicating the regulatory effect of Ca^2+^ on the photosystem. The three-way ANOVA results further confirmed the synergistic effect: the interaction of As×AMF×Ca^2+^ significantly affected *Pn*, *Ci*, *ΦPSII*, and stomatal pore aperture. The combined AMF inoculation and Ca^2+^ application further improved the opening degree of stomata (increased pore aperture), and promoted CO_2_ uptake (increased *Ci*), while more effectively stabilizing the structure and function of *PSII* photosystem (increased *ΦPSII*), thereby maximizing the alleviation of As-induced photosynthetic inhibition.

### 4.3. Enhancement of Osmotic Adjustment and Antioxidant Defense Systems to Alleviate as Stress

As stress induced overaccumulation of reactive oxygen species (ROS) in plants, which is a consequence of photosynthetic destruction, abnormal mitochondrial respiration, and intensified photorespiration [27]. ROS-mediated oxidation of proteins, lipids, and nucleic acids caused severe cell damage or death. However, plants evolved a sophisticated antioxidant defense system, which included antioxidant enzymes (e.g., superoxide dismutase [SOD], peroxidase [POD], and catalase [CAT]), which protected cells and tissues against oxidative damage by efficiently scavenging excess ROS [13]. In this study, As stress reduced the contents of osmolytes (soluble sugar, soluble protein, and proline), and inhibited the activities of antioxidant enzymes (SOD, POD, and CAT), leading to the accumulation of reactive oxygen species (ROS) and subsequent oxidative damage in cotton’s physiological and metabolic processes (Table 5 and Figure 5). Soluble sugars and soluble proteins not only maintained cell turgor but also participated in ROS scavenging and energy metabolism, while proline acts as a versatile protector against osmotic stress and oxidative damage [40]. This reduction in osmolytes may be due to As-induced inhibition of carbohydrate metabolism and protein synthesis, as well as ROS-mediated degradation of osmotic regulators [33]. SOD converted superoxide anions to hydrogen peroxide, and POD/CAT further catalyze the decomposition of hydrogen peroxide into water and oxygen, thereby maintaining cellular redox homeostasis [27]. As stress decreased the activities of antioxidant enzymes, leading to ROS accumulation and lipid peroxidation, which damaged cell membranes and macromolecules [27].

AMF inoculation and exogenous Ca^2+^ application effectively enhanced the capacities of osmotic adjustment and antioxidant defense. AMF-inoculated seedlings exhibited significantly higher contents of the aforementioned osmolytes compared to non-inoculated seedlings under As stress, indicating a crucial role of AMF in enhancing the plant’s osmotic adjustment capacity and alleviating As-induced osmotic stress. AMF promoted the synthesis of osmotic regulators by improving nutrient uptake (e.g., N for protein and proline synthesis) and by regulating the expression of genes related to proline synthesis and sugar metabolism under environmental stress conditions [33,40]. AMF enhanced sugar accumulation by upregulating sucrose phosphate synthase (SPS) activity and reducing acid invertase (AI) activity in host plants [40], while Ca^2+^ stabilized cell membranes and promoted photosynthate conversion to osmolytes [31]. Application of exogenous Ca^2+^ alleviated salt-induced stress of wheat by enhancing the activities of antioxidative enzymes, viz. SOD, POD and CAT activities, respectively, compared with salt-only treatment [17]. The enhanced activities of antioxidant enzymes in AMF-inoculated cotton can be attributed to improved absorption and translocation of water and nutrients (essential for both osmolyte synthesis and antioxidant enzyme activation) and altered host plant gene expression patterns by AMF symbiosis under arsenic (As) stress [14].

Ca^2+^ application enhanced both the accumulation of osmolytes and the activities of antioxidant enzymes in AMF-inoculated and non-inoculated cotton seedlings under arsenic (As) stress. Exogenous Ca^2+^ activated Ca^2+^-dependent protein kinases (CDPKs), which phosphorylate transcription factors regulating osmotic regulator synthesis and antioxidant enzyme expression [15]. It also reduced ROS accumulation by stabilizing cell membranes and inhibiting electron leakage from the electron transport chain [41]. The synergistic effects observed between AMF inoculation and Ca^2+^ application indicated that both factors acted independently yet complementarily—enhancing both the osmotic adjustment capacity and the antioxidant defense system of cotton plants. This finding was consistent with previous reports demonstrating the positive effects of combined AMF and biostimulant treatments on plant stress tolerance [42], highlighting their coordinated role in improving plants’ resilience to As toxicity. Their synergistic effect was evident in significant interactions: leaf soluble sugar is affected by As×Ca^2+^, root soluble sugar by As×AMF and AMF×Ca^2+^, and leaf soluble protein by all pairwise and three-way interactions (As×AMF×Ca^2+^), indicating coordinated regulation of osmolyte synthesis and accumulation (Table 1).

### 4.4. Regulation of Ion Homeostasis to Mitigate as Toxicity

Maintenance of high concentrations of K^+^ and Ca^2+^, and Ca^2+^/K^+^ homeostasis was critical for plant growth and stress tolerance, since these cations played irreplaceable roles in cell membrane stability, enzyme activation, and signal transduction [43,44]. While As stress severely decreased the concentration of K^+^ and Ca^2+^, and disrupted Ca^2+^/K^+^ homeostasis and balance in plant cells [45]. The significant decline in K^+^ and Ca^2+^ contents in cotton leaves and roots under As stress observed in this study, it was consistent with previous findings that As interfered with ion transporters and root nutrient uptake capacity [21,22]. However, AMF inoculation and exogenous Ca^2+^ application effectively alleviated this disruption, highlighting their synergistic and independent regulatory roles in maintaining K^+^ and Ca^2+^ balance under As stress (Figure 7).

AMF inoculation significantly increased K^+^ and Ca^2+^ contents in both cotton leaves and roots under As stress. AMF form extensive extraradical hyphal networks that expand the soil exploration range, enabling cotton to obtain K^+^ and Ca^2+^ ions in soil pores inaccessible to roots alone [43,44]. AMF also regulated the expression of ion transporter genes in host plants: previous studies showed that AMF upregulated K^+^ transporters (e.g., AKT1) and Ca^2+^ channels (e.g., CNGC) to enhance selective absorption of beneficial cations [22,31,46]. AMF improved rhizosphere soil conditions by secreting organic acids and glomalin-related soil proteins (GRSP), which solubilize immobilized K^+^ and Ca^2+^ and reduce As-induced toxicity to root transporters [4,44].

Exogenous Ca^2+^ application not only directly elevated Ca^2+^ and K^+^ content in cotton leaves and roots under As stress, which was consistent with the function of Ca^2+^ as a crucial second messenger in stress response signaling [21,31]. For instance, treatment with Ca(NO_3_)_2_ increased K^+^ and Ca^2+^ concentrations in wheat, particularly cytosolic Ca^2+^ ([Ca^2+^]cyt), thereby enhancing salt stress tolerance [17]. Similarly, Khan et al. (2009) [20] found higher K^+^ and Ca^2+^ levels in mustard plants treated with calcium chloride under salt stress [20]. Ca^2+^ stabilized the cell membrane structure and reduced leakage of K^+^ and other cations under As stress [22]. Exogenous application of an appropriate concentration of Ca^2+^ to broccoli roots under NaCl stress enhanced the uptake of Ca^2+^ and K^+^, and maintained Ca^2+^/K^+^ ion homeostasis in broccoli seedlings [47,48]. Ca^2+^ activated calcium-dependent protein kinases (CDPKs), which in turn regulated the activity of K^+^ transporters and channels, enhancing K^+^ uptake and accumulation [31]. The more pronounced increase in root K^+^ content (28.83% in NM seedlings) compared to leaves (16.36%) suggested that exogenous Ca^2+^ primarily enhanced ion absorption at the root level, which was then translocated to aboveground tissues. Notably, Ca^2+^ application also synergized with AMF to further improve Ca^2+^ and K^+^ contents; however, the increment was slightly lower than that in non-inoculated seedlings, indicating that AMF inoculation and Ca^2+^ application may share partial regulatory pathways in Ca^2+^ and K^+^ acquisition.

The consistent maintenance of a higher Ca^2+^/K^+^ ratio in leaves than in roots in all treatments reflected the distinct physiological requirements of different plant tissues: leaves relied on a higher Ca^2+^/K^+^ ratio to maintain photosynthetic apparatus stability and stomatal function, while roots required a relatively lower ratio for nutrient uptake and cell elongation [43,44]. AMF inoculation and Ca^2+^ application restored the As-disrupted Ca^2+^/K^+^ ratio closer to the Non-stress level, which was crucial for alleviating As-induced physiological disorders.

Two-way ANOVA results revealed specific interactive effects: K^+^ content in roots was affected by the As×Ca^2+^ interaction, indicating that Ca^2+^ regulation of K^+^ acquisition was dependent on intensity of As stress (Table 1). This may be because severe As stress inhibited Ca^2+^ uptake channels, reducing the regulatory efficiency of exogenous Ca^2+^ on K^+^ [31]. Ca^2+^ content in roots was influenced by the As×AMF interaction, suggesting that AMF-mediated Ca^2+^ absorption was enhanced under As stress. This was consistent with the function of AMF in alleviating heavy metal toxicity by enhancing Ca^2+^-dependent membrane stability [22]. The absence of significant interactions on K^+^ in leaves, Ca^2+^ in leaves, and the Ca^2+^/K^+^ ratio in both tissues implied that AMF inoculation and Ca^2+^ application independently regulated these leaf-related ion parameters, providing redundant protection for aboveground tissues critical for photosynthesis. The significant interactions of As×AMF on Ca^2+^ in roots and As×Ca^2+^ on K^+^ in roots confirmed the coordinated regulation of ion uptake and transport by AMF inoculation and Ca^2+^ application. By maintaining stable K^+^ and Ca^2+^ contents and restoring the Ca^2+^/K^+^ ratio, AMF inoculation and Ca^2+^ application ensured the normal function of physiological processes (e.g., stomatal movement, enzyme activity, signal transduction) under As stress. The regulatory effects of AMF inoculation and Ca^2+^ application on K^+^ and Ca^2+^ homeostasis further proved their roles in enhancing cotton’s As tolerance.

## 5. Conclusions

This study aimed to clarify the synergistic mechanisms of AMF inoculation and exogenous Ca^2+^ application on cotton tolerance to arsenic (As) stress by investigating plant growth parameters, root morphology, photosynthetic characteristics, osmotic regulator contents, antioxidant enzyme activities, and ion homeostasis. Among all treatment combinations, the combined application of AMF and exogenous Ca^2+^ (As_100_ + AM + Ca_20_) exhibited the most remarkable ability to alleviate As toxicity, resulting in the best growth performance and physiological tolerance of cotton. Both AMF inoculation and exogenous Ca^2+^ application more synergistically promoted growth parameters (enhanced plant height, shoot and root dry weight), optimized root architecture (increased total root length, root area, and root fork number), improved stomatal function and photosynthetic efficiency (elevated *Pn*, *Ci*, *Fv/Fm*, and *ΦPSII*), enhanced osmotic adjustment capacity (accumulated soluble sugar, soluble protein, and proline), strengthened antioxidant defense systems (increased SOD, POD, and CAT activities), and restored K^+^/Ca^2+^ balance in cotton leaves and roots under As stress. This study demonstrated that AMF inoculation and exogenous Ca^2+^ application enhanced cotton’s As tolerance through improving growth, photosynthetic, metabolic, and ion regulatory processes, which provided novel insights into the synergistic mechanisms of plant-microbe-nutrient interactions in mitigating heavy metal stress. The findings offered a theoretical basis and an environmentally friendly practical strategy for sustainable cotton cultivation in As-contaminated soils.

## Figures and Tables

**Table 1:** Effects of As, AMF and Ca^2+^ and their interactions on cotton growth indicators.

Parameter	As	AMF	Ca^2+^	As×AMF	As×Ca^2+^	AMF×Ca^2+^	As×AMF×Ca^2+^
Plant height	***	***	***	**	***	NS	***
Shoot dry weight	***	***	***	*	NS	NS	**
Root dry weight	***	***	***	NS	NS	NS	NS
Total root length	***	***	***	***	*	NS	NS
Root diameter	***	***	***	NS	NS	NS	NS
Root surface area	***	***	***	***	***	**	*
Root volume	***	***	***	NS	NS	NS	NS
Root branching points	***	***	***	***	***	***	*
Number of root tips	***	***	***	NS	**	NS	NS
*Pn*	***	***	***	***	*	***	**
*gs*	***	***	***	NS	*	NS	NS
*E*	***	***	***	*	NS	NS	NS
*Ci*	***	***	***	NS	NS	*	***
*Φ* *P* *SII*	***	***	***	NS	NS	NS	**
*Fv/Fm*	***	***	***	**	NS	NS	NS
*qP*	***	***	***	NS	NS	NS	NS
NPQ	***	***	***	***	***	NS	NS
Stomatal Length	***	***	***	**	*	NS	NS
Stomatal Width	***	***	***	***	**	NS	**
Pore Aperture	***	***	***	NS	NS	NS	*
Stomatal Density	***	***	***	NS	NS	NS	NS
Soluble sugar content in leaves	***	***	***	NS	***	NS	NS
Soluble sugar content in roots	***	***	***	***	NS	***	NS
Soluble protein content in leaves	***	***	***	***	***	***	***
Soluble protein content in roots	***	***	***	NS	NS	***	NS
Proline in leaves	***	***	***	***	***	NS	**
Proline in Roots	***	***	***	NS	NS	**	NS
SOD in leaves	***	***	***	NS	NS	NS	NS
SOD in roots	***	***	***	NS	NS	NS	NS
POD in leaves	***	***	***	NS	NS	NS	NS
POD in roots	***	***	***	NS	NS	NS	NS
CAT in leaves	***	***	***	NS	NS	NS	NS
CAT in roots	***	***	***	NS	NS	NS	NS
MDA in leaves	***	***	***	NS	NS	NS	NS
MDA in roots	***	***	***	NS	NS	NS	NS
K^+^ content in leaves	***	***	***	NS	NS	NS	NS
K^+^ content in roots	***	***	***	NS	**	NS	NS
Ca^2+^ content in leaves	***	***	***	NS	NS	NS	NS
Ca^2+^ content in roots	***	***	***	*	NS	NS	NS
Ca^2+^/K^+^ in leaves	***	***	***	NS	NS	NS	NS
Ca^2+^/K^+^ in roots	***	***	***	NS	NS	NS	NS

Abbreviations: As: As stress; AMF: *F. mosseae* inoculation; Ca^2+^: exogenous Ca^2+^ application. Note: Significance of three-way ANOVA: NS, not significant; * *p* < 0.05; ** *p* < 0.01; *** *p* < 0.001.

**Table 2:** Effects of AMF and exogenous calcium on net photosynthetic rate *(Pn*), stomatal conductance (*gs*), transpiration rate (*E*), and intercellular CO_2_ concentration (*Ci*) in cotton seedlings under arsenic stress.

As (V) Treatments mg·kg^−1^	Inoculation Treatments	CaCl_2_ Treatments (mmol/L)	Pn (μmol·m^−2^·s^−1^)	gs (mmol·m^−2^·s^−1^)	E (mmol·m^−2^·s^−1^)	Ci (μmol·mol^−1^)
0	NM	0	6.43 ± 0.20 cde	202.67 ± 9.45 c	2.43 ± 0.05 c	307.67 ± 6.11 c
		20	6.84 ± 0.37 cd	230.00 ± 6.56 b	2.80 ± 0.10 b	321.67 ± 5.77 bc
	AM	0	8.24 ± 0.31 b	245.33 ± 3.51 b	2.89 ± 0.07 b	330.33 ± 4.04 b
		20	10.38 ± 0.36 a	279.67 ± 5.13 a	3.21 ± 0.20 a	352.67 ± 3.51 a
100	NM	0	5.45 ± 0.23 f	83.33 ± 5.69 f	1.15 ± 0.08 e	233.33 ± 7.64 f
		20	5.99 ± 0.15 ef	127.00 ± 3.00 e	1.72 ± 0.09 d	273.33 ± 4.73 e
	AM	0	6.25 ± 0.11 de	137.00 ± 5.89 e	1.88 ± 0.02 d	281.00 ± 4.36 de
		20	7.07 ± 0.21 c	179.33 ± 3.21 d	2.25 ± 0.07 c	290.33 ± 5.69 d

Note: Different letters indicate a significant difference at *p* ≤ 0.05. Abbreviations: NM: non-mycorrhizal inoculation; AM: *F. mosseae* inoculation.

**Table 3:** The effects of AMF and exogenous calcium on actual quantum yield in the light-adapted steady state (*ΦPSII*), maximum quantum yield in the dark-adapted state (*Fv/Fm*), photochemical quenching values (*qP*), and nonphotochemical quenching values (*NPQ*) in cotton seedlings under arsenic stress.

As (V) Treatments mg·kg^−1^	Inoculation Treatments	CaCl_2_ Treatments (mmol/L)	*Φ*PSII	Fv/Fm	qP	NPQ
0	NM	0	0.41 ± 0.011 bc	0.81 ± 0.007 bc	0.75 ± 0.009 cd	2.26 ± 0.041 b
		20	0.44 ± 0.007 b	0.82 ± 0.009 bc	0.77 ± 0.004 bc	2.01 ± 0.063 a
	AM	0	0.46 ± 0.013 b	0.83 ± 0.013 ab	0.79 ± 0.009 b	1.93 ± 0.027 c
		20	0.53 ± 0.006 a	0.86 ± 0.010 a	0.82 ± 0.011 a	1.70 ± 0.040 b
100	NM	0	0.24 ± 0.021 f	0.70 ± 0.020 e	0.64 ± 0.008 g	1.55 ± 0.014 de
		20	0.31 ± 0.012 e	0.72 ± 0.003 de	0.67 ± 0.013 f	1.49 ± 0.004 d
	AM	0	0.34 ± 0.016 de	0.76 ± 0.017 d	0.70 ± 0.008 e	1.45 ± 0.023 f
		20	0.37 ± 0.023 cd	0.79 ± 0.007 c	0.73 ± 0.012 de	1.35 ± 0.010 e

Note: Different letters indicate a significant difference at *p* ≤ 0.05. Abbreviations: NM: non-mycorrhizal inoculation; AM: *F. mosseae* inoculation.

**Table 4:** Effects of AMF and exogenous calcium on stomatal length, width, density and pore aperture on cotton leaves under arsenic stress.

As (V) Treatments mg·kg^−1^	Inoculation Treatments	CaCl_2_ Treatments (mmol/L)	Stomatal Length (μm)	Stomatal Width (μm)	Pore Aperture (μm)	Stomatal Density (number·mm^−2^)
0	NM	0	17.29 ± 0.17 c	13.19 ± 0.23 cd	3.54 ± 0.10 cd	339.33 ± 8.50 cd
		20	18.26 ± 0.21 b	13.83 ± 0.06 bc	3.83 ± 0.08 bc	363.00 ± 11.79 bc
	AM	0	18.66 ± 0.15 b	14.46 ± 0.12 b	3.97 ± 0.05 b	390.00 ± 11.36 ab
		20	19.74 ± 0.41 a	16.00 ± 0.41 a	4.46 ± 0.23 a	416.00 ± 23.90 a
100	NM	0	15.49 ± 0.24 e	11.57 ± 0.37 f	2.63 ± 0.11 f	243.33 ± 17.16 f
		20	16.36 ± 0.13 d	12.39 ± 0.21 e	3.04 ± 0.09 e	278.00 ± 14.18 ef
	AM	0	16.58 ± 0.10 d	12.67 ± 0.07 de	3.15 ± 0.04 e	296.67 ± 9.07 e
		20	16.91 ± 0.16 cd	12.88 ± 0.10 de	3.32 ± 0.06 de	313.67 ± 13.05 de

Note: Different letters indicate a significant difference at *p* ≤ 0.05. Abbreviations: NM: non-mycorrhizal inoculation; AM: *F. mosseae* inoculation.

**Table 5:** Effects of AMF and exogenous calcium on the content of osmoregulatory substances of cotton seedlings under arsenic stress.

As (V) Treatments mg·kg^−1^	Inoculation	CaCl_2_ Treatments (mmol/L)	Soluble Sugar Content (mg/g)		Soluble Protein Content (mg/g)		Proline Content (μg/g)	
			Leaf	Root	Leaf	Root	Leaf	Root
0	NM	0	5.24 ± 0.11 c	2.86 ± 0.10 c	6.41 ± 0.13 c	3.60 ± 0.16 c	20.85 ± 0.41 d	20.34 ± 0.48 c
		20	5.81 ± 0.12 b	3.43 ± 0.06 b	8.53 ± 0.05 b	4.93 ± 0.11 b	23.74 ± 0.52 c	23.48 ± 0.33 b
	AM	0	6.76 ± 0.11 a	4.00 ± 0.06 a	8.57 ± 0.09 b	5.10 ± 0.06 ab	25.42 ± 0.31 b	24.44 ± 0.50 ab
		20	7.08 ± 0.16 a	4.25 ± 0.13 a	9.02 ± 0.20 a	5.31 ± 0.06 a	29.78 ± 0.75 a	25.69 ± 0.40 a
100	NM	0	1.93 ± 0.12 g	1.60 ± 0.08 f	3.64 ± 0.16 f	1.06 ± 0.07 f	15.63 ± 0.41 g	13.59 ± 0.93 f
		20	3.02 ± 0.17 f	2.36 ± 0.12 e	4.29 ± 0.09 e	2.25 ± 0.10 e	17.71 ± 0.32 f	15.64 ± 0.31 e
	AM	0	3.48 ± 0.13 e	2.48 ± 0.09 de	4.48 ± 0.11 e	2.46 ± 0.05 de	18.39 ± 0.34 ef	16.44 ± 0.40 e
		20	4.58 ± 0.09 d	2.68 ± 0.08 cd	5.28 ± 0.07 d	2.71 ± 0.07 d	19.57 ± 0.61 de	17.89 ± 0.51 d

Note: Different letters indicate a significant difference at *p* ≤ 0.05. Abbreviations: NM: non-mycorrhizal inoculation; AM: *F. mosseae* inoculation.

## Data Availability

The original contributions presented in this study are included in the article. Further inquiries can be directed to the corresponding author.
